# QTL mapping of wheat plant architectural characteristics and their genetic relationship with seven QTLs conferring resistance to sheath blight

**DOI:** 10.1371/journal.pone.0174939

**Published:** 2017-04-06

**Authors:** Yan Guo, Ziyi Du, Jiang Chen, Zhongjun Zhang

**Affiliations:** Plant Pathology Department, College of Plant Protection, China Agricultural University, Beijing, China; Institute of Genetics and Developmental Biology Chinese Academy of Sciences, CHINA

## Abstract

Sheath blight is one of the most devastating wheat diseases worldwide. Breeding resistant cultivars is the most powerful strategy to defeat the disease. Plant resistance on “disease escape” works through modulation of morphological traits and shows sustainable resistance to disease. Plant architectural traits have been reported to play a significant role in disease response. Therefore, exploring the genetic relationship between plant architecture and disease resistance is of importance to the understanding of plant resistance via “disease escape”. Using an F_9_ population of 266 RILs (Recombinant Inbred Lines) derived from the cross of Luke × AQ24788-83, we have generated a linkage map of 631 markers on 21 chromosomes. In this study, we present the QTL identification of fourteen plant architectural characteristics and heading time from two years and analyze their genetic relationships with seven previously published QTLs to sheath blight (QSBs, *QSe*.*cau*), including plant height (PH), the space between the flag leaf and penultimate leaf (fdR), heading date (Hd), and other traits. Twelve stable QTLs of the morphological traits were identified with good consistency across five replicates. For the seven previously published QSBs, we found no significant association with plant height. However, some of the QSBs displayed strong associations with plant architectural traits and heading date. Especially, *Q*_*fdR*_.*cau-1AS*, *Q*_*Hd*_.*cau-2BS*, *Q*_*fdR*_.*cau-5DL*, and *Q*_*fdR*_.*cau-6BL* were respectively mapped to the same regions as *QSe*.*cau-1AS*, *QSe*.*cau-2BS*, *QSe*.*cau-5DL*, and *QSe*.*cau-6BL*. Taken together, we have demonstrated that plant height did not exert a direct influence on the resistance to sheath blight conferred by the seven QSBs and that the plant architecture and heading date did exhibit a tight relationship with the resistance. Therefore, this study provides a novel evidence to help understand sheath blight resistance in wheat. In addition, the linked morphological characteristics and the generated flanking markers will facilitate breeding for resistance to sheath blight in wheat.

## Introduction

Wheat (*Triticum aestivum* L.) is one of the top three crops worldwide, feeding more than 35% of the world’s population [1]. Sheath blight (caused by *Rhizoctonia cerealis*), also known as sharp eyespot disease, is a major limiting factor in wheat yield and grain quality. To date, no immunity gene against sheath blight has been found [2]. Nonetheless, many cultivars with moderate resistance to sheath blight have been reported [3]. In addition, a number of quantitative trait loci (QTLs) for resistance to sheath blight (QSBs) have been identified in genetic linkage maps [4–6]. However, few of them have been applied to breeding practices, which have been constrained by limited understanding of the genetic relationship between plant resistance to sheath blight and agronomic characteristics to some extent [7].

It has been hypothesized that plants require and exhibit resistance to fungal disease through either physiological resistance or disease escape [8]. Srinivasachary et al. showed that disease escape represents an important group of disease resistances [9]. Disease escape resistance works by modulating plant canopy morphological traits to lessen disease expansion, avoids the rapid generation of new virulent races by releasing the stress of plant-pathogen physiological interactions and shows more sustainable resistance. Sheath blight, a soil-borne fungal disease, starts by invading the basal sheath of the plant stem and then transfers upwards towards healthy tissues. Multiple environmental factors can affect the spread of sheath blight, such as the wind, rain, planting density and plant morphological characteristics. Plant architecture varies greatly among different genotypes and exerts a crucial influence on the microclimate of plants, such as contact frequency, light transmittance, humidity, and temperature. These canopy parameters have been shown to play a significant role in sheath blight epidemics [10–12]. It was also documented that the effective management of crop architectural structures provides a potential strategy for sheath blight control [13]. Therefore, the study of the genetic relationship between QSBs and plant architectural traits will be beneficial to our understanding of the QTL resistance mechanisms along with further deployment into breeding programs [7].

Several studies on sheath blight resistance in rice have identified QSBs in the same regions as the QTLs for plant height or heading time [14–16]. However, no correlation was observed between wheat sheath blight resistance and plant height or heading time in other studies [17]. Sharma et al. speculated that sheath blight resistance was not directly affected by plant height [18]. Another study hypothesized that some plant resistances function indirectly through adjusting canopy capacity, tissue contact and leaf wetness [19].

Similarly, in our wheat genetic breeding program, we have reported seven QTLs to sharp eyespot disease (*QSe*.*cau-1AS*, *QSe*.*cau-2BS*, *QSe*.*cau-3BS*, *QSe*.*cau-4AL*, *QSe*.*cau-5DL*, *QSe*.*cau-6BL*, and *QSe*.*cau-7BL*), none of which showed statistically significant associations with plant height [6]. In this study, we hypothesize that several components of wheat plant architecture and canopy structure, such as the space between the flag leaf and penultimate leaf, have a close relationship with sheath blight resistance in wheat. In addition, heading time has been occasionally reported to have a significant influence on sheath blight resistance in rice [17]. Therefore, the main objectives of this work were to conduct QTL mapping of 14 wheat architectural characteristics and heading dates and to study their genetic relationship with the seven previously identified QSBs in a linkage map, in order to more accurately facilitate the understanding of disease resistance mechanisms and the practical application to breeding for disease resistance in wheat.

## Materials and methods

### Plant materials

A winter wheat cross of Luke × AQ24788-83 was developed in May 2002, and 1,580 recombination inbred lines (RILs) were created and numbered. Luke (Pedigree: PI 178383/2*Burt//CItr13438) is a winter wheat cultivar from the National Small Grain Collection, Aberdeen, ID83210, USA. AQ24788-83 (AQ) was selected and advanced to the F_14_ generation from the progeny of a double-cross among four Chinese wheat landraces of Mazhamai/Baiqimai//Hongqimai/Qingshoumai [20]. Luke is shorter and highly resistant to sheath blight and has a later heading time, while AQ is taller and susceptible to sheath blight and has an earlier heading time [6]. A population consisting of 266 randomly selected lines from the original RIL populations was used to conduct QTL mapping and to analyze the genetic relationship between disease resistance and morphological characteristics.

### Field trials

Field trials were conducted in Beijing during two wheat growing seasons. The 266 F_9_ and F_10_ RILs were cultivated at China Agricultural University Experimental Station in Shangzhuang (39°54′20” N, 116°25′29” E, Beijing) in a randomized complete block design with two replicates in the 2011–2012 growing season (2012) and with three replicates in the 2012–2013 growing season (2013). An individual plot consisted of a single row 80 cm in length sown with 20 seeds of a RIL, and the adjacent plots were 40 cm apart. Within each replicate, three plots each of the parents Luke and AQ were included. To avoid the influence of sheath blight disease on morphological features, we did not inoculate the isolates of the sheath blight pathogen, and there was no obvious disease observed in the fields.

**Fig 1:**
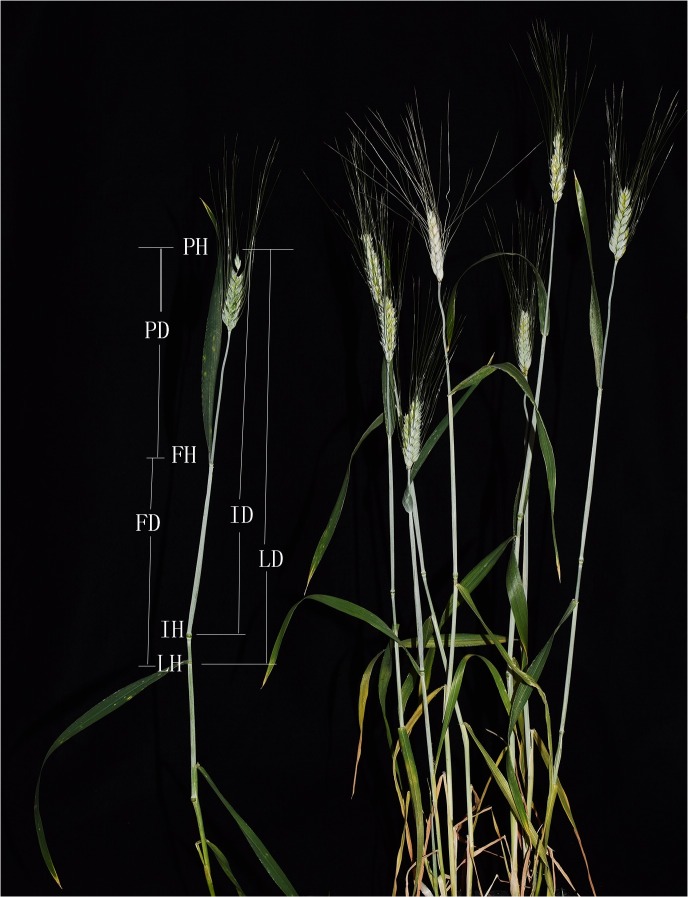
Architecture of the Flag Leaf, the Uppermost Internode, and the Penultimate Leaf of Wheat Plants.

According to the positions of the major leaves and nodes from top to bottom as shown in Fig 1, we investigated 12 wheat plant architectural characteristics in all the plots in both years, including plant height with spike (PH, cm), the flag leaf height (FH, cm), the uppermost internode height (IH, cm), the penultimate leaf height (LH, cm), the distance between the plant top and the flag leaf ear (PD = PH-FH, cm), the distance between the flag leaf and the penultimate leaf (FD = FH-LH, cm), the distance between the plant top and the uppermost internode (ID = PH-IH, cm), the distance between the plant top and the penultimate leaf (LD = PH-LH, cm), and the ratios of PD, FD, ID, LD to plant height (pdR, fdR, idR, and ldR, %). The peak of sheath blight basically occurred when plants headed out in each growing season [21]. At heading time, the flag leaf appears upright, and the penultimate leaf seemed to affect the canopy conditions of plants to some extent. Heading date (Hd), the angle between the penultimate leaf ear and stem (Ag, 0°-90°) and flag leaf length (FLL, cm) were also measured. These 15 characteristics of each RIL were measured in three plants, and mean values were calculated to represent the characteristics of each RIL.

### Statistical analysis

Microsoft Excel 2010 and IBM SPSS Statistics 19 (IBM Co., USA) were used to conduct statistical analyses of the morphological trait data from the 2012 and 2013 seasons. For each morphological characteristic, the values of the two replicates in 2012 and three replicates in 2013 were averaged, respectively, to display the statistical characterization of the RILs and the parents.

### Genetic linkage mapping

The 266 RILs of Luke × AQ were used to construct a genetic linkage map. DNA of Luke, AQ, and the RILs was extracted using the cetyl trimethyl ammonium bromide (CTAB) method as described [22]. There were 2,012 pairs of SSR and EST-SSR primers selected to screen for the polymorphisms between Luke and AQ. The sequences of these markers were obtained from the public domain, including BARC, CFA, CFB, CFD, EST-SSR, GDM, GPW, and GWM (http://www.scabusa.org, http://wheat.pw.usda.gov, http://wheat.pw.usda.gov). PCRs were conducted with the Applied Biosystems GeneAmp PCR system 9700 (Applied Biosystems Inc., Foster City, USA) in volumes of 20 μl containing 1 unit Taq DNA polymerase, 2 μl 10× PCR buffer, 1.8 mM MgCl_2_, 0.3 mM dNTP, 10 M of each primer and 30 ng template DNA with the following condition: 95°C for 3 minutes, followed by 38 cycles of 95°C for 30 seconds, 50–65°C for 45 seconds, 72°Cfor 30 seconds, and a final extension for 10 minutes. PCR products were separated on 6% denaturing polyacrylamide gels and imaged with the silver staining method [23]. The markers with clear and reproducible bands were finally chosen to genotype RILs for genetic map construction.

The software MAPMAKER/EXP3.0 was used to construct the linkage map [24]. The Kosambi function and three-point tests were applied. The map frame was established initially with COMPARE, ORDER, and MAP commands. Then, we used TRY command to add additional markers to extend the linkage map. Other similar parameters and procedures reported previously were adopted [6,25]. An individual group was built with the adjacent markers less than 50 cM apart.

Additionally, QTL mapping was performed with ICIMapping version 4.0.6.0 software. Composite Interval Mapping (CIM) was used with the standard model at a logarithm of the odds (LOD) threshold of 2.5. The 15 characteristics of the five replicates (2012 and 2013) were averaged initially to conduct QTL mapping in the linkage map. Then, all the replicates of the traits were individually applied to perform genetic mapping to test the consistency of the QTLs among different replicates and seasons.

## Results

### Statistical characterization of traits investigated

There is a distinct structure of wheat plants with major organs, including the flag leaf, the uppermost internode, and the penultimate leaf (Fig 1). From the top to the bottom of a wheat plant, the flag leaf and the penultimate leaf divide the wheat plant into the three parts: PD, FD and LH. The whole plant was also partitioned into spike-neck (ID) and internodes (IH) by the uppermost node and partitioned into LD and LH by the penultimate leaf. It can be seen that PH = PD+FD+LH = ID+IH = LD+LH. In addition, the ratios of PD, FD, ID and LD to plant height (pdR, fdR, idR and ldR) were also applied to represent wheat architectural characteristics.

**Fig 2:**
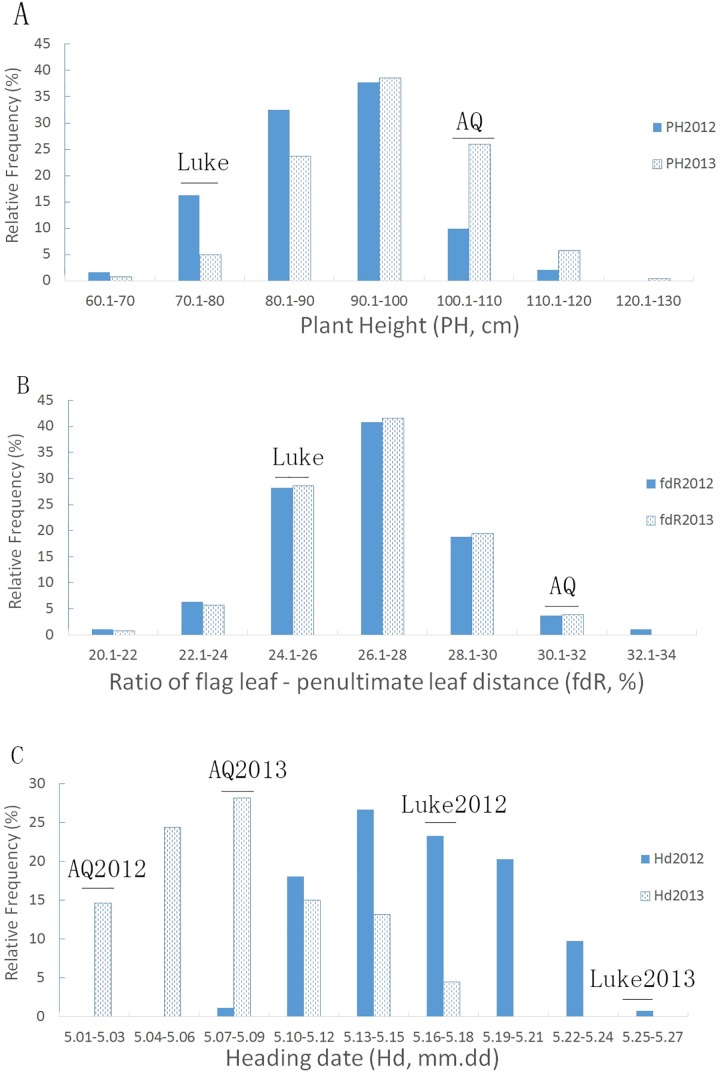
Distribution of Three Plant Morphological Traits for 266 RILs. **(**A) Distribution of plant height (PH). (B) Distribution of the ratio of flag leaf—penultimate leaf to plant length (fdR). (C) Distribution of heading date (Hd).

For the 15 morphological characteristics, the two replicates of 2012 and the three replicates of 2013 were averaged for each year. As shown in Fig 2, the 14 plant architecture traits and heading dates of the RILs and the parents varied greatly. The plant heights of the RILs ranged from 65.83 cm to 113.33 cm in 2012 and from 62.67 cm to 121.67 cm in 2013. For fdR, the RILs had a range of 20.26% to 33.58% in 2012 and of 21.28 to 31.30% in 2013. However, there was a large deviation of the heading dates between 2012 (5.09–5.24) and 2013 (5.01–5.17). The PH, fdR and heading time were normally distributed with considerable ranges. Specifically, the population in 2013 was taller in plant length and headed out nine days earlier than in 2012. In addition, the RILs showed a significant transgressive segregation in the morphological traits compared to the parents. As seen in Fig 1 and Fig 2, plant height and its components constitute a distinct hierarchy of plant canopy structure, which contributes to the microenvironment of the development of wheat plants and sheath blight disease.

### Construction of the genetic linkage map

In the established genetic map, there were 606 SSR markers, 1 EST marker and 6 DarT markers, which were located on the 21 chromosomes of wheat by referring to `previously published wheat maps. The numbers of markers on each chromosome ranged from 18 (on chromosome 6A) to 53 (on chromosome 3B). The distances between the adjacent markers were all less than 45.9 cM. The whole linkage map spanned 4,432.7 cM, with the shortest length of 104 cM on chromosome 6A and longest length of 282 cM on chromosome 2D. The average distance between adjacent markers was 7.2 cM.

### QTL mapping of morphological characteristics

**Fig 3:**
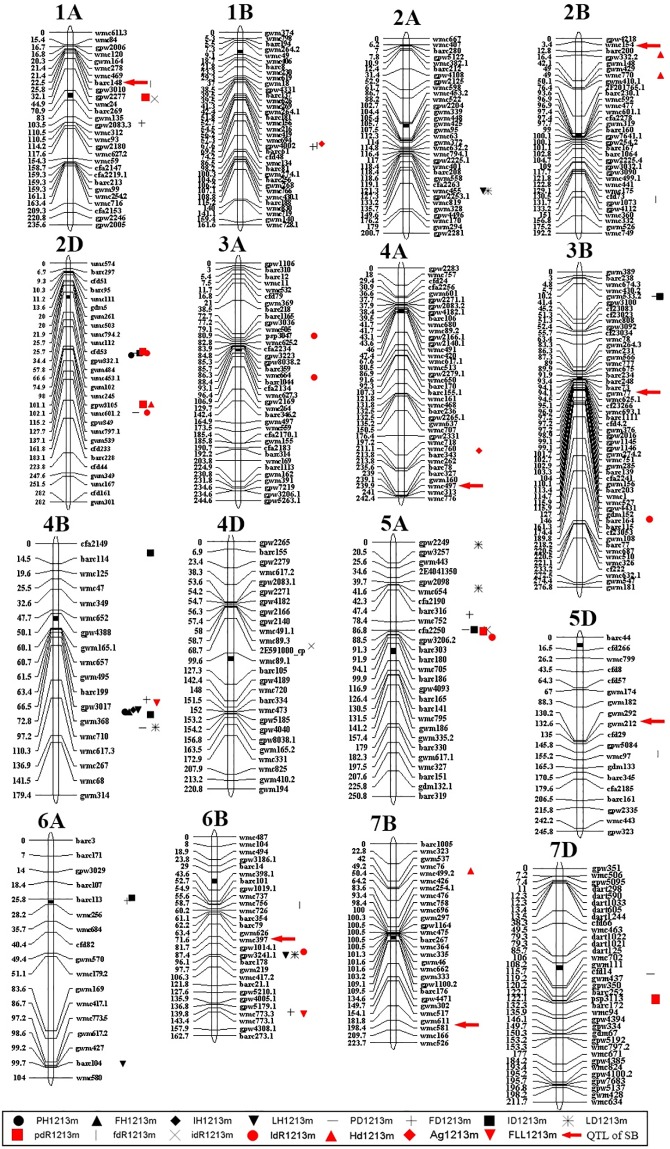
QTL Map of 15 Morphological Characteristics and Published Sheath Blight Resistance.

Using the average values of the five replicates of the RILs, a total of 59 QTLs were identified on 16 chromosomes in the genetic linkage map (Fig 3). Among these QTLs, eight QTLs for the heights (PH, FH, IH, and LH) were identified on chromosomes 2A, 2D, 4B, 6A, and 6B, which accounted for 13.6% of all the QTLs. There were 23 QTLs for the distances (PD, FD, ID, and LD) mapped on 10 chromosomes, and 20 QTLs for the proportions (pdR, fdR, idR, and ldR) on 11 chromosomes. In addition, four QTLs for heading time, two QTLs for stem-leaf angle, and two QTLs for flag leaf length were detected on chromosomes 1B, 2B, 2D, 4A, 4B, 6B, and 7B. In terms of LOD values, 43% of the QTLs were significant at the level of a LOD threshold over 5.0.

Importantly, several QTLs for the canopy traits showed a close relationship with the QSBs in the linkage map. There were one to four QTLs for morphological traits in the same region as the QSBs, except for *QSe*.*cau-3BS* and *QSe*.*cau-7BL*. In the interval between barc148 and wmc24 on chromosome 1A, three QTLs for fdR, pdR, idR overlapped near *QSe*.*cau-1AS*. The LOD values ranged from 2.88 to 5.29, and they could explain 4%– 7% of the phenotype variance. On chromosome 2B, two QTLs for heading date were mapped right at the position of *QSe*.*cau-2BS*. The QTL for heading date had a significant LOD value (8.14), which could explain 17% of the phenotype variance. For *QSe*.*cau-3BS*, the QTLs for PD, ID, and ldR located on chromosome 3B were all over 50 cM apart. Still, there was one QTL for the angle of stem-leaf (Ag) in the same region as *QSe*.*cau-4AL* flanked by wmc760 and wmc776. One QTL for fdR was identified at the position of gwm212 on chromosome 5D, which was followed by gpw5087 and *QSe*.*cau-5DL*. At the same time, *QSe*.*cau-6BL* was detected in the interval between the QTL for fdR and the QTLs for LH, LD and ldR. The two QTLs for fdR on 5D and 6B had LOD values of 4.74 and 11.47, which could explain 6% and 15% of the phenotype variance, respectively. On chromosome 7B, only one QTL for Hd was mapped on the short arm and was far away from *QSe*.*cau-7BL*.

Of the 59 QTLs, there were twelve QTLs mapped consistently across the five replicates in the 2012 and 2013 seasons, which were designated *Q*_*fdR*_.*cau-1AS*, *Q*_*Hd*_.*cau-2BS*, *Q*_*PH*_.*cau-4BL*, *Q*_*FH*_.*cau-4BL*, *Q*_*IH*_.*cau-4BL*, *Q*_*LH*_.*cau-4BL*, *Q*_*FD*_.*cau-4BL*, *Q*_*ID*_.*cau-4BL*, *Q*_*LD*_.*cau-4BL*, *Q*_*fdR*_.*cau-5DL*, *Q*_*fdR*_.*cau-6BL*, and *Q*_*Hd*_.*cau-7BS* (Table 1). Of these stable QTLs, three QTLs for fdR were mapped on chromosomes 1AS, 5DL and 6BL, two QTLs for Hd on 2BS and 7BS, and 7 QTLs for PH, FH, IH, LH, FD, ID, and LD were mapped in the same interval between barc199 and wmc710 on 4BL. Additionally, *Q*_*fdR*_.*cau-1AS*, *Q*_*Hd*_.*cau-2BS*, *Q*_*fdR*_.*cau-5DL*, and *Q*_*fdR*_.*cau-6BL* were mapped in the same regions as four published cases of QSBs (*QSe*.*cau-1AS*, *QSe*.*cau-2BS*, *QSe*.*cau-5DL*, and *QSe*.*cau-6BL*). In addition, *Q*_*Hd*_.*cau-7BS* was flanked by gwm537 and wmc426 and showed a significance level of 5.0–5.9 in five replicates but was mapped at the other distal end away from *QSe*.*cau-7BL*.

**Table 1:** Stable QTLs for Morphological Traits across Five Replicates and the Related QSBs.

Chromosome	QTL for sheath blight resistance			QTL for morphological trait				
	QSB	Nearest marker	Position/cM	QTL	Marker region	Position/cM	LOD value	Variance (%)
1A	*QSe*.*cau-1AS*	barc148	22	*Q*_*fdR*_	barc148-wmc120	22–25	3.8–5.3	7–11
2B	*QSe*.*cau-2BS*	wmc154	12	*Q*_*Hd*_	barc200-gem410.1	21–22	5.6–6.7	8–13
4B	_	_	_	*Q*_*PH*_	barc199-gwm368	65–70	5.2–8.0	13–18
	_	_	_	*Q*_*FH*_	barc199-wmc710	65–80	6.7–10.6	13–22
	_	_	_	*Q*_*IH*_	barc199-wmc710	65–83	4.3–8.5	8–18
	_	_	_	*Q*_*LH*_	barc199-wmc710	65–81	3.7–7.0	7–17
	_	_	_	*Q*_*FD*_	barc199-wmc710	64–79	4.5–8.1	7–20
	_	_	_	*Q*_*ID*_	gpw3017-wmc710	68–86	1.9–6.1	6–16
	_	_	_	*Q*_*LD*_	barc199-wmc710	64–82	1.7–3.9	4–14
5D	*QSe*.*cau-5DL*	gwm212	135	*Q*_*fdR*_	gwm292-wmc97	150–155	0.9–4.5	4–7
6B	*QSe*.*cau-6BL*	wmc397	72	*Q*_*fdR*_	Wmc756-wmc726	56–58	4.1–5.9	8–15
7B	*QSe*.*cau-7BL*	wmc581	198	*Q*_*Hd*_	gwm537-wmc426	46–50	5.0–5.9	7–9

## Discussion

To explore the genetic relationship between the seven QSBs (*QSe*.*cau-1AS*, *QSe*.*cau-2BS*, *QSe*.*cau-3BS*, *QSe*.*cau-4AL*, *QSe*.*cau-5DL*, *QSe*.*cau-6BL*, and *QSe*.*cau-7BL*) and plant architecture, fifteen characteristics, investigated from five replicates in two growing seasons, were used for QTL mapping to study their positional correlation with the QSBs in a linkage map. A number of QTLs for the morphological traits were identified and overlapped with each other, including plant architectural traits, heading time, and QSBs.

It has been reported in rice QSB studies that QSBs had been frequently identified to link with plant height or heading time [7,26]. However, Sharma et al. speculated that sheath blight resistance was not directly affected by plant height [18]. The results of this study suggest a similar conclusion as none of the seven published cases of QSBs showed significant associations with plant height statistically and genetically. Still, the plant architectural traits were shown to link with some QSBs in the genetic map (Fig 3). In addition, an increased capacity of the plant canopy was suggested to promote greater contact of plant tissues and accelerate disease extension by retaining moisture and leaf wetness [19]. Therefore, the study of the genetic mapping of plant architectural traits and the seven published QSBs can provide further evidence to help understand the disease escape mechanisms of sheath blight resistance.

**Fig 4:**
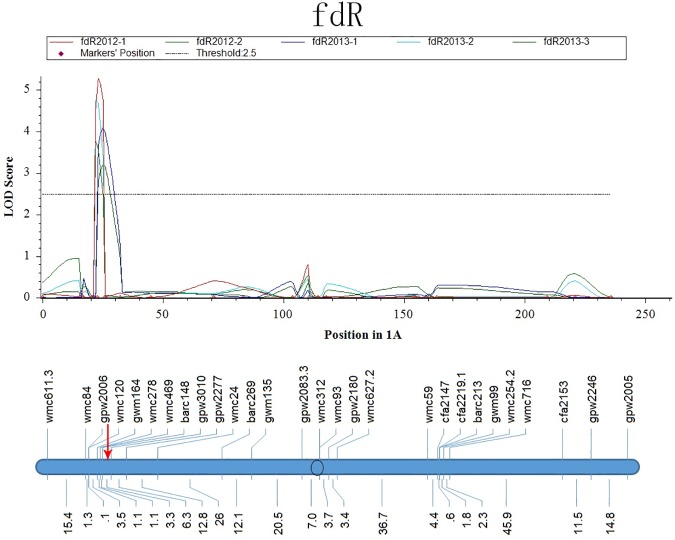
LOD Curves of QTLs for the Ratio of Flag Leaf -Penultimate Leaf (fdR) on Chromosome 1A. The red arrow represents the position of *QSe*.*cau-1AS*.

For *QSe*.*cau-1AS* on the short arm of chromosome 1A, three QTLs for pdR, fdR, and idR were identified in the same region as *QSe*.*cau-1AS* with the mean values of the five replicates (Fig 3). In particular, *Q*_*fdR*_.*cau-1AS* was consistent across five replicates and was mapped at marker barc148, the nearest maker to *QSe*.*cau-1AS* (<2.0 cM) (Fig 4). The RILs with a smaller ratio of the flag leaf-penultimate leaf to plant length (fdR) have broader basal spaces, which was less conducive to conditions of disease development, including humidity maintenance, light transmittance, and temperature. Moreover, a larger area of plant base was not advantageous for plant contact (leaf to leaf, leaf to sheath) and pathogen spread. Therefore, it can be suggested that fdR has large effects on canopy traits and microclimates of plant and disease growth and, in turn, works in the disease escape mechanism of *QSe*.*cau-1AS* resistance. Still, further study is needed to verify whether *Q*_*fdR*_.*cau-1AS* and *QSe*.*cau-1AS* come from the same allele with a pleiotropic effect or different loci tightly linked together. Extending plant space will bring yield loss in wheat production, although it was revealed to be a good method to control the disease. If the QSB and the plant architecture QTL are different loci, more populations and markers will be needed to separate them to facilitate the future application of the QSB. If they prove to be the same allele, the introgression of the disease resistance would result in yield loss from spreading canopy. For the latter condition, we need to consider balancing the plant architecture feature and yield characteristics in wheat practical breeding. Additionally, the combination of multiple disease resistance QTLs, low tiller number genes and high-yield genes will be an effective strategy to avoid yield loss in wheat.

It has been reported that wheat sheath blight and rice sheath blight are caused by similar pathogens, *R*. *cerealis* and *R*. *solani*. There might be some similarities and associations between the sheath blight resistance of wheat and rice. Based on wheat-rice comparative genomics, R5 of rice is the homologous chromosome to wheat 1AS. Four QTLs to sheath blight have been reported on the R5, while no association was detected with plant height or heading time [7,27]. In this study, the identification of *Q*_*fdR*_.*cau-1AS*, co-located with *QSe*.*cau-1AS*, provides a potential clue into the genetic correlation between QSBs and plant architecture in wheat and rice. Because there is some pathogenicity difference between *Rhizoctonia* spp. strains of rice and wheat, more work will be required to test the hypothesis of the relationship between rice QSBs and wheat QSBs [28].

**Fig 5:**
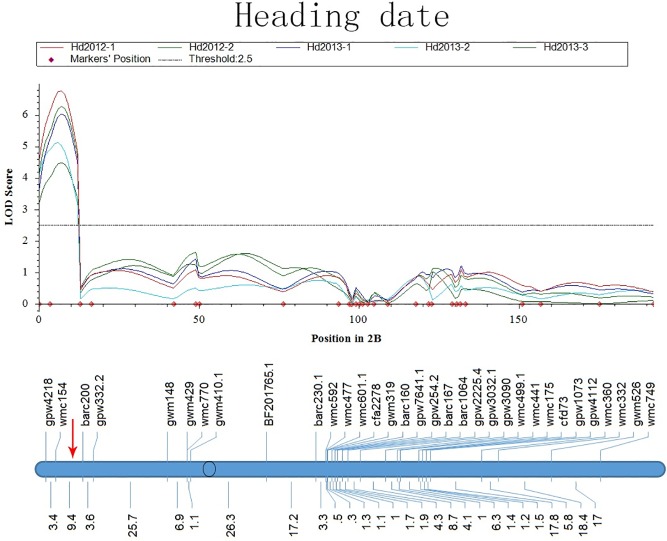
LOD Curves of QTLs for Heading Date (Hd) on Chromosome 2B. The red arrow represents the position of *QSe*.*cau-2BS*.

On chromosome 2B, *Q*_*Hd*_.*cau-2BS* was mapped in the interval between barc200 and gdm410.1 and was within 10.0 cM of *QSe*.*cau-2BS* (Fig 5). Some studies on rice QSBs revealed that heading date has a significant influence on the threat of sheath blight [12]. A number of rice QSBs cases were reported to be co-localized with QTL for heading time [14,29,30]. It was explained that varieties with a later heading time and a longer duration cycle avoided the climate conditions of the disease occurrence and spread [7,31]. Additionally, a later heading date could help strengthen the tightness of the stem closing by the sheath, where sheath blight disease begins to invade the plants [6]. Although it is unknown whether *Q*_*Hd*_.*cau-2BS* and *QSe*.*cau-2BS* come from one allele with a pleiotropic effect or distinctive loci tightly linked, delaying heading time was an important method to avoid sheath blight. However, cultivars with late heading time will go against breeding needs in wheat [32]. On the homologous chromosome of R7S in rice, there was one QSB (Rh7) mapped and linked tightly with one QTL for heading time [7,15].

In the region of *QSe*.*cau-4AL*, one QTL for the stem-leaf angle (Ag) was located in the interval between wmc718 and barc78 with the mean of the five replicates (Fig 3). It has been reported that leaf angle has some effect on sheath blight intensity [9]. Stem-leaf angle has an effect on tissue contact and canopy conditions, including light transmittance, aeration, and humidity among plants. As the QTL for Ag did not show consistency across all five replicates, we need more information to further validate its existence. Still, the co-location of the stem-leaf angle with QSB could offer some clue in the association between sheath blight resistance and the stem-leaf angle.

**Fig 6:**
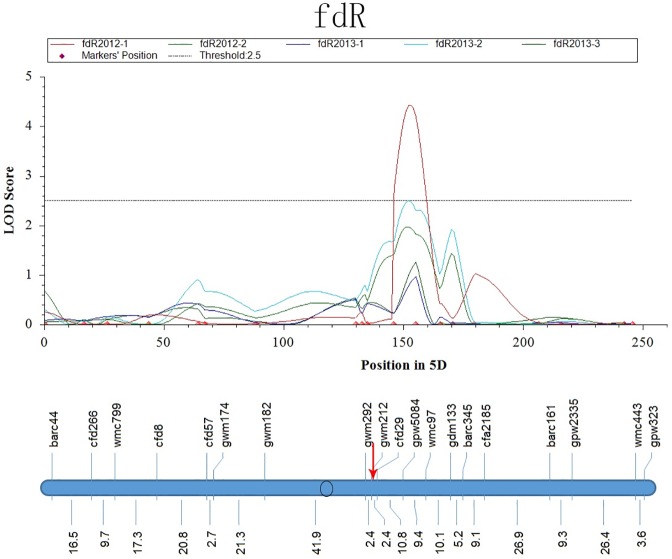
LOD Curves of QTLs for the Ratio of Flag Leaf -Penultimate Leaf (fdR) on Chromosome 5D. The red arrow represents the position of *QSe*.*cau-5DL*.

**Fig 7:**
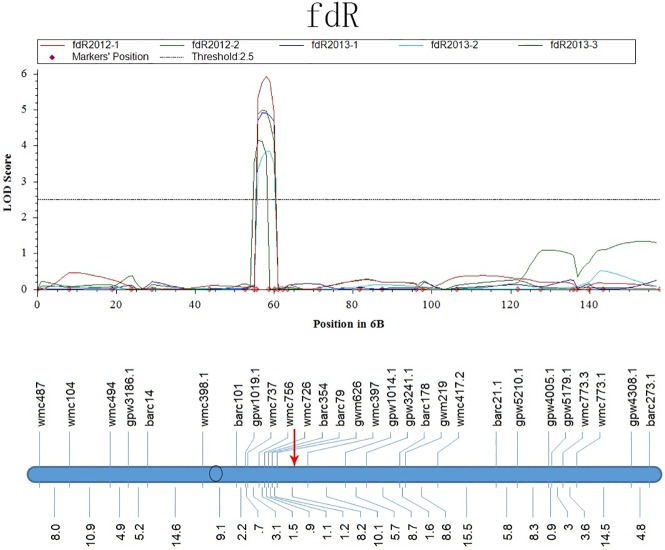
LOD Curves of QTLs for the Ratio of Flag Leaf -Penultimate Leaf (fdR) on Chromosome 6B. The red arrow represents the position of *QSe*.*cau-6BL*.

In the region of *QSe*.*cau-5DL*, *Q*_*fdR*_.*cau-5DL* was mapped to the interval between gpw5084 and wmc97 across the five replicates (Fig 6). *Q*_*fdR*_.*cau-5DL* and *QSe*.*cau-5DL* were tightly linked within a 10.0 cM region between cfd29 and gpw5084. For *QSe*.*cau-6BL*, five QTLs for the morphological traits fdR, FD, LH, LD and ldR were mapped as neighboring *QSe*.*cau-6BL* with the mean values of the five replicates. In particular, the QTL for fdR (*Q*_*fdR*_.*cau-6BS*) showed good stability across the five replicates with phenotype variance of 8% to 15% (Fig 7). Thus, the region of gpw1019.1-barc79 indicated a complex interaction between disease resistance and multiple plant architecture traits [33]. In addition, our data also suggested that fdR might exert some influence on the resistance of *QSe*.*cau-5DL* and *QSe*.*cau-6BL* as described for *QSe*.*cau-1AS*.

**Fig 8:**
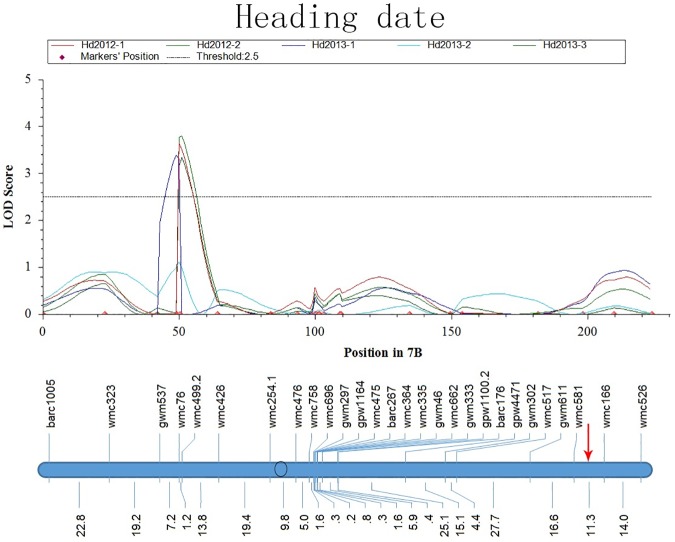
LOD Curves of QTLs for Heading Date (Hd) on Chromosome 7B. The red arrow represents the position of *QSe*.*cau-7BL*.

On chromosome 7B, *Q*_*Hd*_.*cau-7BS* was detected consistently on the end of the short arm, while *QSe*.*cau-7BL* was located on the other end of the chromosome (Fig 8), indicating that *Q*_*Hd*_.*cau-7BS* and *QSe*.*cau-7BL* have no correlation in genetics. By comparison to the rice chromosome R6S in homology, one rice QSB was identified and overlapped a QTL for heading date. However, more research will be required to test whether these two QSBs and the QTL for heading date have some connection in their origins.

**Fig 9:**
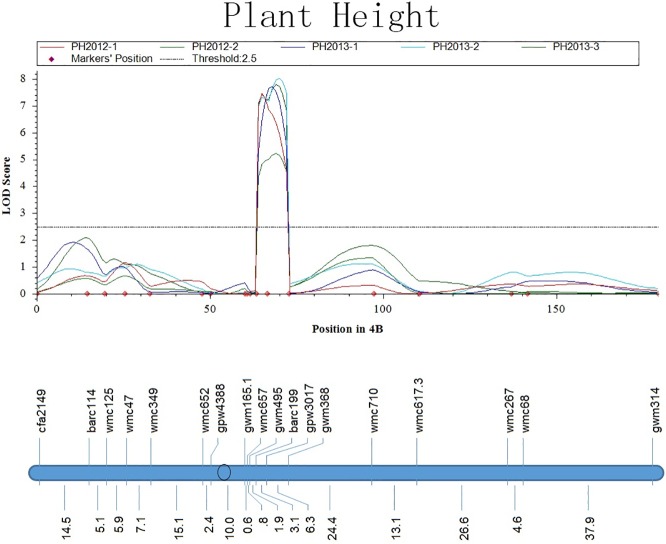
LOD Curves of QTLs for Plant Height (PH) on Chromosome 4B.

Additionally, one major QTL for plant height, *Q*_*PH*_.*cau-4BL*, was mapped in the interval between barc199 and wmc710, a region where no resistance QTL for sheath blight was detected consistently (Fig 9). Meanwhile, the other six stable QTLs (*Q*_*FH*_.*cau-4BL*, *Q*_*IH*_.*cau-4BL*, *Q*_*LH*_.*cau-4BL*, *Q*_*FD*_.*cau-4BL*, *Q*_*ID*_.*cau-4BL*, and *Q*_*LD*_.*cau-4BL*) overlapped at the same position as *Q*_*PH*_.*cau-4BL* (Table 1). With similar LOD curves, it is likely that these seven QTLs, or at least some of them, come from one individual major gene with a strong pleiotropic effect. To the best of our knowledge, *Q*_*PH*_.*cau-4B* is apparently different from any dwarfing gene or semi-dwarfing gene on 4B, such as *Rh1* and *Rh3*, and lies in one gene-rich region between the markers wmc657 and wmc617 [34,35]. Previous studies have shown that one resistance gene against Ug99 races of stem rust was identified in this region, as well as one resistance QTL to common bunt disease (caused by *Tilletia tritici* Wint and *T*. *laevis* Kühn), which was also co-located with a QTL for plant height [36,37]. It is unknown whether the gene for the Ug99 resistance and the QTL for common bunt resistance have any relationship with the plant canopy traits in this region. In this study, no significant association was observed between wheat plant height and sheath blight resistance, although some rice QSBs have been reported to be co-localized with QTLs for plant height [26].

This study provides a new genetic evidence for the hypothesis that plant architecture plays a potential role in the resistance to sheath blight by adjusting the basal micro-climate of plants. We also verified that plant height does not exert a direct effect on sheath blight resistance in wheat [18]. By comparing to rice QSBs in the homologous regions, we have revealed that wheat resistance QTLs have different genetic associations with morphological traits from rice. Moreover, the clustered QTLs exhibit a diversified and distinctive interaction between disease resistance and multiple morphological characteristics, which provides novel insights into the understanding of sheath blight resistance mechanisms and the deployment of the resistance genes in wheat breeding practices.

## Supporting information

S1 Table Statistical Result of 15 Morphological Traits of 266 RILs and the Parents. (DOCX)
