# Supplementary material for: QTL mapping of wheat plant architectural characteristics and their genetic relationship with seven QTLs conferring resistance to sheath blight
Source: PLoS One. 2017 Apr 6;12(4):e0174939. doi: 10.1371/journal.pone.0174939 (PMC5383044; doi:10.1371/journal.pone.0174939)
Supplement: S1 Table — (DOCX) [file pone.0174939.s001.docx]

**S1 Table.Statistical Results of 15 Morphological Traits of 266 RILs and the Parents.**

|  |  |  | 2012RIL | | | |  | 2013RIL | | | |  | Parent | |
| --- | --- | --- | --- | --- | --- | --- | --- | --- | --- | --- | --- | --- | --- | --- |
| Trait | Name | Count | Min | Max | Mean | SD |  | Min | Max | Mean | SD |  | Luke | AQ |
| Height (cm) | PH | 266 | 65.83 | 113.33 | 89.34 | 9.70 |  | 62.67 | 121.67 | 95.45 | 9.89 |  | 73.40 | 105.00 |
|  | FH | 266 | 47.83 | 85.17 | 66.28 | 7.69 |  | 46.00 | 97.33 | 73.07 | 7.75 |  | 58.20 | 80.00 |
|  | IH | 266 | 33.17 | 69.00 | 49.82 | 6.46 |  | 31.67 | 74.67 | 55.01 | 6.55 |  | 41.40 | 60.33 |
|  | LH | 266 | 28.17 | 60.33 | 42.49 | 5.73 |  | 29.00 | 66.33 | 47.65 | 5.88 |  | 39.40 | 47.67 |
| Distance (cm) | PD | 266 | 11.33 | 33.50 | 23.04 | 4.01 |  | 11.67 | 33.33 | 22.38 | 4.16 |  | 15.20 | 25.00 |
|  | FD | 266 | 17.17 | 31.00 | 23.76 | 2.87 |  | 17.00 | 33.00 | 25.42 | 2.96 |  | 18.80 | 32.33 |
|  | ID | 266 | 27.33 | 51.67 | 39.48 | 4.92 |  | 25.33 | 53.00 | 40.44 | 5.10 |  | 32.00 | 44.67 |
|  | LD | 266 | 33.00 | 61.33 | 46.86 | 5.85 |  | 31.33 | 64.00 | 47.80 | 6.24 |  | 34.00 | 57.33 |
| Ratio(%) | pdR | 266 | 14.90 | 32.37 | 25.77 | 3.45 |  | 14.57 | 31.58 | 23.38 | 3.35 |  | 20.45 | 23.84 |
|  | fdR | 266 | 20.26 | 33.58 | 26.65 | 2.01 |  | 21.28 | 31.30 | 26.69 | 1.85 |  | 25.81 | 30.80 |
|  | idR | 266 | 34.34 | 52.40 | 44.25 | 3.39 |  | 33.84 | 50.56 | 42.41 | 3.24 |  | 43.50 | 42.57 |
|  | ldR | 266 | 42.50 | 59.72 | 52.46 | 3.53 |  | 38.34 | 59.43 | 49.81 | 3.70 |  | 46.27 | 54.64 |
| Heading date | Hd | 266 | 5.09 | 5.25 | 5.16 | 0.04 |  | 5.01 | 5.17 | 5.08 | 0.04 |  | 5.23 | 5.12 |
| Stem-leaf angle (°) | Ag | 266 | 18.00 | 42.75 | 28.64 | 5.08 |  | 22.67 | 35.67 | 28.86 | 2.66 |  | 25.00 | 30.00 |
| Flag leaf length(cm) | FLL | 266 | 13.22 | 26.38 | 19.10 | 2.06 |  | 12.83 | 25.67 | 17.47 | 2.26 |  | 18.56 | 16.22 |
